# The therapeutic potential and mechanisms of targeting the PTBP1/Nogo-A/NgR axis in PTSD induced by single prolonged stress in mice

**DOI:** 10.1038/s12276-025-01629-4

**Published:** 2026-01-21

**Authors:** Bing-Yao Liu, Xing-Dong Chen, Hui-Lin Liu, Si-Wei Wang, Qian-Zhong Song, Hui Cheng, Sen Li, Hai-Yan Wang, Xiu-Min Lu, Yong-Tang Wang

**Affiliations:** 1 State Key Laboratory of Trauma and Chemical Poisoning, Daping Hospital, Army Medical University, Chongqing, China; 2 College of Pharmacy and Bioengineering, Chongqing University of Technology, Chongqing, China

**Keywords:** Post-traumatic stress disorder, Post-traumatic stress disorder

## Abstract

The pathophysiology of post-traumatic stress disorder (PTSD) shows notable associations with compromised hippocampal neurophysiology. Notwithstanding ongoing debates, PTBP1 knockdown (KD) demonstrates the capacity to drive glia-to-neuron reprogramming, potentially offering therapeutic benefits for some neurodegenerative pathologies. However, PTBP1 KD can upregulate the expression of Nogo-A by alternative splicing, triggering the inhibition of nerve regeneration. Currently, the role of PTBP1 in PTSD remains unknown. Here we sought to elucidate the neurorestorative effects of modulating the PTBP1/Nogo-A/NgR axis in a mouse model of PTSD established through the single prolonged stress paradigm, and the mechanisms were further investigated through a series of experiments including pathological and molecular detection. The results indicated that PTBP1 KD ameliorates PTSD-like behaviors in mice by balancing Bcl-2/Bax expression and suppressing Caspase-3 splicing activation to inhibit hippocampal neuronal apoptosis, enhancing synaptic plasticity through upregulating PSD95 and SYN1, increasing dendritic spine density and stabilizing axonal architecture via elevated NF200 expression. However, compared with single prolonged stress alone, PTBP1 KD potentiates the activation of Nogo-A/NgR pathway, adversely impacting both dendritic morphology and axonal elongation. Therefore, we proposed a combined KD of PTBP1 and NgR to counteract the adverse effects mediated by Nogo-A signal activation, effectively promoting dendritic growth and axonal extension in hippocampal neurons of PTSD mice. Our findings underscore the potential and limitations of PTBP1 as a therapeutic target and propose a novel method for PTSD treatment through combined target intervention of PTBP1 and NgR. This study provides a theoretical foundation for multitarget intervention strategies in the treatment of PTSD and related disorders.

## Targeting PTBP1 and NgR promotes recovery in PTSD mice

Post-traumatic stress disorder (PTSD) is a mental health condition that can develop after experiencing a traumatic event. This study investigates the role of a protein called PTBP1 in PTSD. Researchers used a type of virus to reduce PTBP1 levels in the brains of mice with PTSD-like symptoms. They found that lowering PTBP1 improved the mice’s behavior, reducing fear and anxiety. Results showed that reducing PTBP1 helped protect brain cells from dying and improved connections between neurons. However, it also activated a pathway that could hinder nerve growth. To address this, the researchers also targeted another protein, NgR, which helped counteract the negative effects. In conclusion, targeting PTBP1 and NgR together may offer a new approach to treating PTSD by improving brain function while minimizing side effects.

This summary was initially drafted using artificial intelligence, then revised and fact-checked by the author.

## Introduction

Post-traumatic stress disorder (PTSD) is a severe psychiatric condition typically developing after exposure to major traumatic events. It is clinically characterized by persistent hyperarousal of traumatic memories, profound fear/anxiety and impairments in learning and cognitive functions^1,2^. In recent years, the global occurrence of PTSD has shown an upward trend^3,4^. Although the exact pathophysiology remains incompletely characterized, PTSD symptoms are closely linked to persistent reactivation of fear memory engrams. The hippocampus serves as a pivotal center for fear memory consolidation and stress response regulation, whereas patients with PTSD demonstrate hippocampal structural and functional abnormalities, including reduced volume, neuronal loss (predominantly apoptosis), impaired synaptic plasticity and disrupted axonal regeneration^5–7^.

Numerous studies have attempted to identify effective therapeutic targets for PTSD, yet with limited success. In recent years, polypyrimidine tract-binding protein 1 (PTBP1) has garnered increasing attention due to its pivotal role in regulating neuronal growth and development^8–10^. PTBP1, a multidomain member of the PTB protein family, contains four RNA recognition motifs flanked by nuclear localization/export signals. As a splicing regulator, PTBP1 modulates alternative splicing dynamically in both nuclear and cytoplasmic compartments through targeting the 3′-untranslated region (UTR) UC-rich sequence in pre-mRNAs. Beyond splicing, PTBP1 coordinates mRNA stability, polyadenylation and translational regulation across developmental stages^11,12^. PTBP1 exhibits high expression levels in neural precursor cells and undergoes gradual downregulation as cellular differentiation progresses. By contrast, PTBP2 displays neural-specific expression, with its expression levels transiently upregulating following the downregulation of PTBP1 during neurodevelopment, followed by subsequent downregulation^13,14^.

Previous studies demonstrate that PTBP1 knockdown (KD) alone sufficiently induces mature cells transdifferentiating into functional neurons, revealing its therapeutic potential for neurodegenerative diseases^15–17^. Despite ongoing debates, this strategy confers benefits such as improved behaviors, suggesting that PTBP1 downregulation may ameliorate disease phenotypes through alternative mechanisms^18,19^. Indeed, PTBP1 extensively participates in neuronal-specific mRNA splicing events, thereby modulating the physiological and pathological conditions of neurons.

On the one hand, PTBP1 plays a crucial role in the generation and survival of neurons. During the differentiation of mouse embryonic stem cells into neuronal progenitor cells, PTBP1 targets the seventh exon of pre-B-cell leukemia homeobox transcription factor 1 (PBX1) to inhibit its expression. PBX1 has been linked to critical developmental processes and serves as a key regulator of neuronal lineage gene transcriptional activation. This suggests that PTBP1 expression during the neuronal progenitor cell stage restricts neuronal lineage gene activation, with its dysregulation potentially contributing to various disorders^8,20^. Notably, PTBP1 downregulation within physiological limits promotes normal neuronal development, whereas its excessive suppression induces premature differentiation of neural stem cells^21^. Other studies have demonstrated a critical association between PTBP1 and neuronal apoptosis regulation. During neurodevelopment, PTBP1 downregulation mediates exon 5 inclusion of Bak1 through splice-specific regulatory mechanisms, thereby suppressing Bak1 expression to inhibit neuronal apoptosis^22^. On the other hand, PTBP1 regulates synaptic plasticity and axonal regeneration. Synaptic Ras GTPase-Activating Protein 1 (SynGAP1), a Ras GTPase-activating protein, facilitates synaptic maturation and plasticity by directly binding to postsynaptic density protein 95 (PSD95)^10,23^. During the initial stages of neurodevelopment, PTBP1/2 facilitates the inclusion of the alternative splice site within intron 10 of SynGAP1, thereby mediating nonsense-mediated mRNA decay of SynGAP1 protein. In addition, PTBP1 directly inhibits the splicing of exon 18 of PSD95, leading to frameshift reading of its mRNA, premature translation termination and, subsequently, nonsense-mediated mRNA decay, which ultimately suppresses the expression of PSD95. These events are detrimental to neuronal synaptic plasticity^10,24^. Fibroblast growth factor receptor 2 (FGFR2) is indispensable in the processes of myelination, dendrite outgrowth and adult neurogenesis^25,26^. PTBP1 suppresses the inclusion of FGFR2 exon IIIb during splicing, thereby inhibiting FGFR2 expression. Although direct evidence for this splicing regulation occurring ubiquitously in neural tissues is lacking, given the enrichment of PTBP1 and FGFR2 in brain tissue, particularly in glial cells, it is speculated that PTBP1 may be detrimental to the structural integrity of neuronal cells mediated by FGFR2^27,28^. In conclusion, downregulation of PTBP1 expression may represent an effective strategy to promote neuronal growth and survival under pathological conditions.

It is noteworthy that, despite the beneficial effects of PTBP1 downregulation on neuronal growth and survival, alterations in the expression levels of some of its effector targets appear to be detrimental to the survival state of neurons. Nogo-A, a potent myelin-associated inhibitor belonging to one of the three splice variants of the RTN4 (Nogo) family, exhibits the strongest inhibitory capacity toward axonal regeneration among the Nogo family members^29,30^. As a common receptor for myelin-associated inhibitors, the Nogo receptor (NgR), forms a complex with p75NTR/TROY-LINGO-1, thereby permitting the transduction of extracellular inhibitory signals into the cell. These signals activate downstream ROCK2 kinase (or other ROCK isoforms) via RhoA, subsequently triggering actin depolymerization and the activation of downstream signaling pathways. This signaling cascade modulates hippocampal synaptic plasticity, induces growth cone collapse and impairs axonal regeneration^31,32^. PTBP1 regulates the skipping mutation of exon 3 of RTN4 through alternative splicing, thereby modulating the expression of Nogo-A. The majority of studies suggest that downregulation of PTBP1 induces an upregulation of Nogo-A^33–35^. Consequently, even though the targeted KD of PTBP1 facilitates a shift in genes toward splice variants that promote neuronal growth through alternative splicing, the downregulation of PTBP1 may concurrently activate Nogo/NgR inhibitory signaling targeting axonal regeneration, adversely impacting synaptic plasticity and inhibiting axonal regeneration.

In summary, downregulation of hippocampal PTBP1 may rescue neuronal loss and synaptic plasticity impairments, stabilizing neuronal structure and positioning it as a potential therapeutic target for PTSD and other neurological disorders. The aim of this study was to investigate the effects and underlying mechanisms of downregulating hippocampal PTBP1 expression on the behavior of PTSD mice. Furthermore, this study focuses on potential detrimental effects of PTBP1 suppression-induced Nogo/NgR signaling hyperactivation in PTSD mice, while developing therapeutic strategies to mitigate these compensatory pathway responses.

## Materials and methods

### Animals

In total, 201 female C57BL/6J mice (specific pathogen-free, 8-week-old, body weight ~20 g) were sourced from Beijing Spaf Biotechnology and were maintained in individually ventilated cages at a density of five per cage with ad libitum access to food and water. Environmental conditions were controlled at 21–23 °C and 50–60% relative humidity under a 12-h/12-h light–dark cycle (illumination 7:00–19:00). An acclimation period of 2 weeks was allowed for the mice. All animal experiments were approved by the Laboratory Animal Welfare and Ethics Committee of Third Military Medical University (Army Medical University) (approval number AMUWEC 20210966) and strictly adhered to the ‘Guide for the Care and Use of Laboratory Animals’ (2006), published by the Ministry of Science and Technology of China.

### Plasmids and viruses

Two distinct single guide RNA sequences target the *PTBP1* gene: 5′-TGTAGATGGGCTGTCCACGAAGCACTGGCG-3′ and 5′-GCTTGGAGAAGTCGATGCGCAGCGTGCAGC-3′. A single guide RNA sequence specifically designed for the *NgR* gene is 5′-GTGCCCGGCAGTCACACACCCAGGGGTTGT-3′. In this study, adeno-associated virus (AAV) packaging services were provided by Obio Technology. The PTBP1-targeting virus was designated AAV-PTBP1-HA, with a titer of 2.43 × 10¹² vg/ml, and the blank control virus AAV-Empty-HA lacks the targeting sequences. Similarly, the NgR-targeting virus is AAV-NgR-FLAG, with a titer of 2.11 × 10¹³ vg/ml, and its blank control is AAV-Empty-FLAG without the NgR-targeting sequence.

### Single prolonged stress

The single prolonged stress (SPS) paradigm is the most classical stress induction method for investigating PTSD^36^. Initially, mice were individually confined in a cylindrical restraint (5 cm inner diameter, 15 cm deep, partially closed bottom, with a stopcock to limit movement) in darkness for 2 h without food or water while ensuring free respiration. Following this, they were subjected to forced swimming in freshwater (20 cm deep, 23–25 °C) for 20 min. After 15 min of rest, the mice were deeply anesthetized with ether vapor, and after awakening, the mice were reared normally in the cage.

### Lateral ventricle injection

Mice were anesthetized with 1% pentobarbital sodium (60 mg/kg) and immobilized using a stereotaxic apparatus. After disinfection, surgery was performed to expose the skull. The virus, diluted to 5 × 10^9 ^vg/μl in phosphate-buffered saline (PBS), was injected into the lateral ventricle (coordinates: anterior–posterior −1 mm, medial–lateral −1.7 mm, dorsal–ventral −2 mm from the dura) at a rate of 0.1 μl/min using a Hamilton syringe with a 32 G needle^37^. The needle was left in place for 5 min after injection.

### Experimental groups

First, to verify the KD efficiency of PTBP1 and NgR, as well as the key effector molecules baseline impact of PTBP1 KD on normal mice, we injected normal mice with AAV-PTBP1 and AAV-NgR, and the corresponding blank viruses into the lateral ventricle, and used western blot analysis to detect the expression of PTBP1, NgR and other molecules in the hippocampus (*n* = 3 per group). Then, to investigate the behavioral impacts and underlying mechanisms of PTBP1 KD in normal and PTSD mice, we randomly allocated mice into five groups (*n* = 12 per group): the control group (normal control), the AAV-PTBP1 group (received AAV-PTBP1-HA virus injection into the lateral ventricle only), the AAV-PTBP1/SPS group (received AAV-PTBP1-HA virus injection post-SPS modeling), the AAV-Empty/SPS group (received AAV-Empty-HA virus injection post-SPS modeling) and the SPS group (SPS modeling only). To further investigate the combined effects of PTBP1 and NgR KD on neuronal synaptic plasticity and axonal regeneration, we randomly established five groups (*n* = 9 per group): control, AAV-NgR/SPS, AAV-PTBP1+NgR/SPS, AAV-Empty/SPS and SPS. Specifically, the AAV-NgR/SPS group underwent an injection of AAV-NgR-FLAG virus into the lateral ventricle post-SPS modeling, the AAV-PTBP1+NgR/SPS group received a co-injection of AAV-PTBP1-HA and AAV-NgR-FLAG viruses post-SPS modeling, and the AAV-Empty/SPS group received a co-injection of AAV-Empty-HA and AAV-Empty-FLAG viruses post-modeling. The remaining groups were handled as previously described. To investigate the impact of combined PTBP1 and NgR KD on the baseline levels of key effector molecules in normal mice, the animals were randomly assigned to two groups (*n* = 3 per group): the AAV-Empty group and the AAV-PTBP1+NgR group, receiving only AAV injection. Lastly, to compare the behavioral effects of combined PTBP1 and NgR KD versus PTBP1 KD alone on PTSD and normal mice, we randomly established six groups (*n* = 13 per group): control, AAV-PTBP1+NgR, AAV-PTBP1/SPS, AAV-PTBP1+NgR/SPS, AAV-Empty/SPS and SPS, with each group being treated as previously outlined.

### Open field test

The open field test (OFT) evaluates spontaneous locomotor activity and exploration behavior of animals in novel environments, quantifying motor function and anxiety-related responses. The apparatus consists of a box-shaped field (50 × 50 × 40 cm) with flooring divided into a 5 × 5 grid. The central 3 × 3 grid (nine zones) and four corner quadrants (four zones) constitute defined observational areas. During testing, subjects are randomly placed facing away from the experimenter in the corner. Automated tracking records behavioral metrics over a 5-min session: total distance, the number of crossing, number of crossing in the central zone, time spent in the central zone and time spent in the corner zone.

### Elevated plus maze

The elevated plus maze (EPM) is used to assess anxiety in experimental mice. The maze consists of two open arms (70 × 6 cm) and two enclosed arms (70 × 6 × 20 cm), arranged in a cruciform shape with a central intersection, and the entire structure is elevated 55 cm above the ground. During testing, a mouse is randomly selected and placed in the central area facing away from the experimenter, and the behavior tracking system is immediately activated. Behavioral parameters analyzed within 5 min include: number of entries into open arm, time spent in open arm, time spent in enclosed arm, the percentage of open arm entries (OE%), the percentage of time spent in open arm (OT%) and anxiety index (calculated as 1 − (OT%/2 + OE%/2)). These metrics collectively evaluate the mouse’s locomotor activity and anxiety state.

### Morris water maze

The Morris water maze (MWM) test serves as an instrument for assessing spatial memory and learning abilities in mice. It comprises a circular pool and a submerged platform. The pool has a diameter of 120 cm and a height of 40 cm, and the water temperature is maintained at 23–25 °C. The pool is divided into four quadrants with distinct markers placed in its east, south, west and north directions. The platform, with a diameter of 8 cm, is positioned in the third quadrant, located 1–2 cm beneath the water surface. The experiment is divided into two phases: place navigation and spatial probe. During the place navigation phase (days 1–6), mice are placed into the water facing the pool wall from the marker site nearest to the platform, allowing them to search for the platform. If a mouse fails to find the platform within 1 min, it is guided onto the platform. The mouse is then allowed to remain on the platform for 20 s for learning and memory consolidation, followed by a 20-s rest period. Afterward, the mouse is moved clockwise to the next marker site for repeated trials, with two sites being moved sequentially each day, totaling three trials per day. From day 2 onward, the starting site for each mouse is shifted clockwise by one site compared with the previous day, with other procedures remaining the same as on day 1. Over these 6 days, the time taken for the mouse to find the platform is recorded, referred to as escape latency. During the spatial probe test phase (day 7), the platform is removed, and the mouse is placed at the farthest position from the platform for a 1-min free exploration. The duration of time the mouse spends in the third quadrant (time spent in the target quadrant) and the number of times it crosses the original platform location (number of crossing platform) are recorded.

### Pathological staining

Hippocampal pathological analysis was conducted using hematoxylin–eosin (HE) and Nissl staining. Mice were deeply anesthetized via intraperitoneal injection of 1% sodium barbital (60 mg/kg) and subsequently transcardially perfused with ice-cold 0.01 M PBS, followed by 4% paraformaldehyde (PFA) at 4 °C. Brains were dissected, post-fixed in 4% PFA at 4 °C for 24 h and subsequently processed through graded ethanol dehydration, chloroform clearing and paraffin embedding. Coronal sections (5 μm thickness) were obtained using a microtome (Thermo Scientific, cat. no. KD-3368AM) and stored at room temperature. Before staining, sections underwent xylene deparaffinization and ethanol gradient rehydration, followed by staining with HE (Solarbio, cat. nos. G4070 and G1100) and self-configuring Nissl stain. Hippocampal cellular morphology was systematically evaluated under an optical microscope microscope (Olympus CX33). HE staining reveals uniform chromatin distribution in normal nuclei, while damaged nuclei exhibit hyperchromatic condensation with chromatin aggregation. Apoptotic cells demonstrate nuclear fragmentation and the presence of apoptotic bodies. In Nissl staining, normal nuclei appear light blue, whereas injured nuclei appear dark blue.

### TUNEL staining

Terminal deoxynucleotidyl transferase dUTP nick-end labeling (TUNEL) staining was performed using a commercial kit (Beyotime, cat. no. C1090). Image acquisition was performed using a Nikon ECLIPSE Ti laser confocal microscope (Nikon), and image analysis was performed via Image-Pro Plus 5.0 (Media Cybernetics).

### Golgi-Cox staining

The rapid Golgi staining kit (FD Neuro Technologies, cat. no. PK-401) was used to investigate hippocampal synaptic plasticity in this study. The brain tissues of mice were washed with precooled 0.01 M PBS. Tissues were then prepared according to kit instructions and frozen for embedding. Brain sections (100 μm in thickness) were cut using a freezing microtome (Leica, cat. no. CM1950) and placed on glass slides precoated with 1% gelatin. Then, the staining was performed according to the procedure of the kit. The morphology of hippocampal neurons was observed using an optical microscope (Olympus, cat. no. CX33), and the images were processed using ImageJ software (ImageJ, cat. no. 1.51n).

### Immunofluorescence staining

In this study, cryopreserved brain tissue sections were utilized for immunofluorescence analysis. Mice were deeply anesthetized by intraperitoneal administration of 1% sodium barbital (60 mg/kg), followed by transcardial perfusion with ice-cold 0.01 M PBS (pH 7.4) and 4% PFA. After post-fixation in 4% PFA at 4 °C for 24 h, the brain was dehydrated twice using 30% sucrose solution and ultimately embedded in OCT embedding agent and stored at −80 °C. Tissue sections of 10 μm thickness were prepared using a Leica CM1950 cryostat and stored at −80 °C. Tissue sections were equilibrated to room temperature, followed by washes with 4 °C PBS (pH 7.4), then were blocked with 5% bovine serum albumin (containing 0.3% Triton X-100, pH 7.4) for 1 h at room temperature. After a brief PBS rinse, sections were incubated with primary antibodies at 4 °C overnight. On the next day, sections were washed with PBS, followed by incubation with appropriate fluorescent secondary antibody at room temperature for 1 h, then washed again, and anti-fluorescence quenching agent containing DAPI (Beyotime, cat. no. P0131) was adopted. Finally, the plates were sealed with nail oil. Image acquisition was carried out using a Nikon ECLIPSE Ti laser confocal microscope (Nikon). The mean fluorescence intensity of the obtained immunofluorescence images was analyzed by ImageJ software (ImageJ, cat. no. 1.51n). Primary antibodies included anti-HA-tag (1:200, abcam, ab18181), anti-FLAG (1:100, abcam, ab205606), anti-NeuN (1:200, Millipore, MAB377), anti-Bcl-2 (1:200, Sigma, SAB4500003), anti-PSD95 (1:500, CST, 36233s), anti-SYN1 (1:500, abcam, ab254349), anti-GAP-43 (1:200, CST, 8945s) and anti-NF200 (1:200, abcam, ab8135). Fluorescent secondary antibodies included 488 conjugated donkey anti-mouse antibody (1:200, Jackson, 715-545-150), 488 conjugated donkey anti-rabbit antibody (1:200, Jackson, 711-545-152), Cy3 conjugated donkey anti-rabbit antibody (1:200, Jackson, 711-165-152) and Cy3 conjugated Alpaca anti-mouse antibody (1:200, Jackson, 615-165-214).

### Western blot analysis

Fresh hippocampal tissue was dissected from mice, and total protein was extracted immediately. Proteins of various molecular weights were separated by sodium dodecyl sulfate–polyacrylamide gel electrophoresis. Electrophoresis was performed at 60 V (stacking gel) and 120 V (resolving gel). Subsequently, the proteins were transferred to polyvinylidene fluoride membranes (0.2 μm, Millipore, cat. no. ISEQ00010; or 0.45 μm, Millipore, cat. no. IPVH00010). The membranes were blocked with 5% nonfat milk at room temperature for 1 h. Primary antibodies, including anti-PTBP1 (1:2,000, Abcam, ab133734), anti-RTN4R (1:7,000, Boster, A02250-2), anti-NeuN (1:1,000, Millipore, MAB377), anti-Bcl-2 (1:1,000, Sigma, SAB450003), anti-Bax (1:1,000, CST, 14796S), anti-Caspase-3 and anti-Cleaved Caspase-3 (1:1,000, Abcam, ab13847), anti-PSD95 (1:7,000, CST, 36233S), anti-SYN1 (1:7,000, Boster, BA1421), anti-NF200 (1:1,000, Abcam, ab8135), anti-GAP-43 (1:1,000, CST, 8945S), anti-Nogo-A (1:1,000, Abcam, ab47085), anti-RhoA (1:1,000, CST, 2117S), anti-ROCK2 (1:1,000, CST, 9029S), anti-β-actin (1:7,000, Sigma, A1978) and anti-GAPDH (1:7,000, CST, 5174S), were incubated with the membranes overnight at 4 °C. The membranes were then washed three times with TBST (10 min each). Secondary antibodies, including rabbit anti-mouse (1:7,000, Bioss, bs-0296R-HRP) and goat anti-rabbit (1:7,000, Bioss, bs-0295G-HRP), were incubated with the membranes at room temperature for 1 h and washed again three times with TBST (10 min each). Finally, an appropriate amount of chemiluminescent detection reagent (Millipore, cat. no. WBKLS0500) was dispensed, and detection was carried out using an imaging system (Vilber, cat. no. Fusion-solo 6.0). Image processing was then performed using ImageJ software (ImageJ, cat. no. 1.51n).

### Statistical analysis

All experimental data were analyzed using GraphPad Prism 9.0 (GraphPad Software), and the results were expressed as mean ± standard error of the mean (s.e.m.). Comparisons between two groups were conducted using the *t*-test, while data from three or more groups were analyzed by one-way or two-way analysis of variance, followed by Tukey’s post-hoc test to assess differences among groups. *P* < 0.05 was considered statistically significant.

## Results

### Identification of PTBP1 KD in vivo

**Fig. 1:**
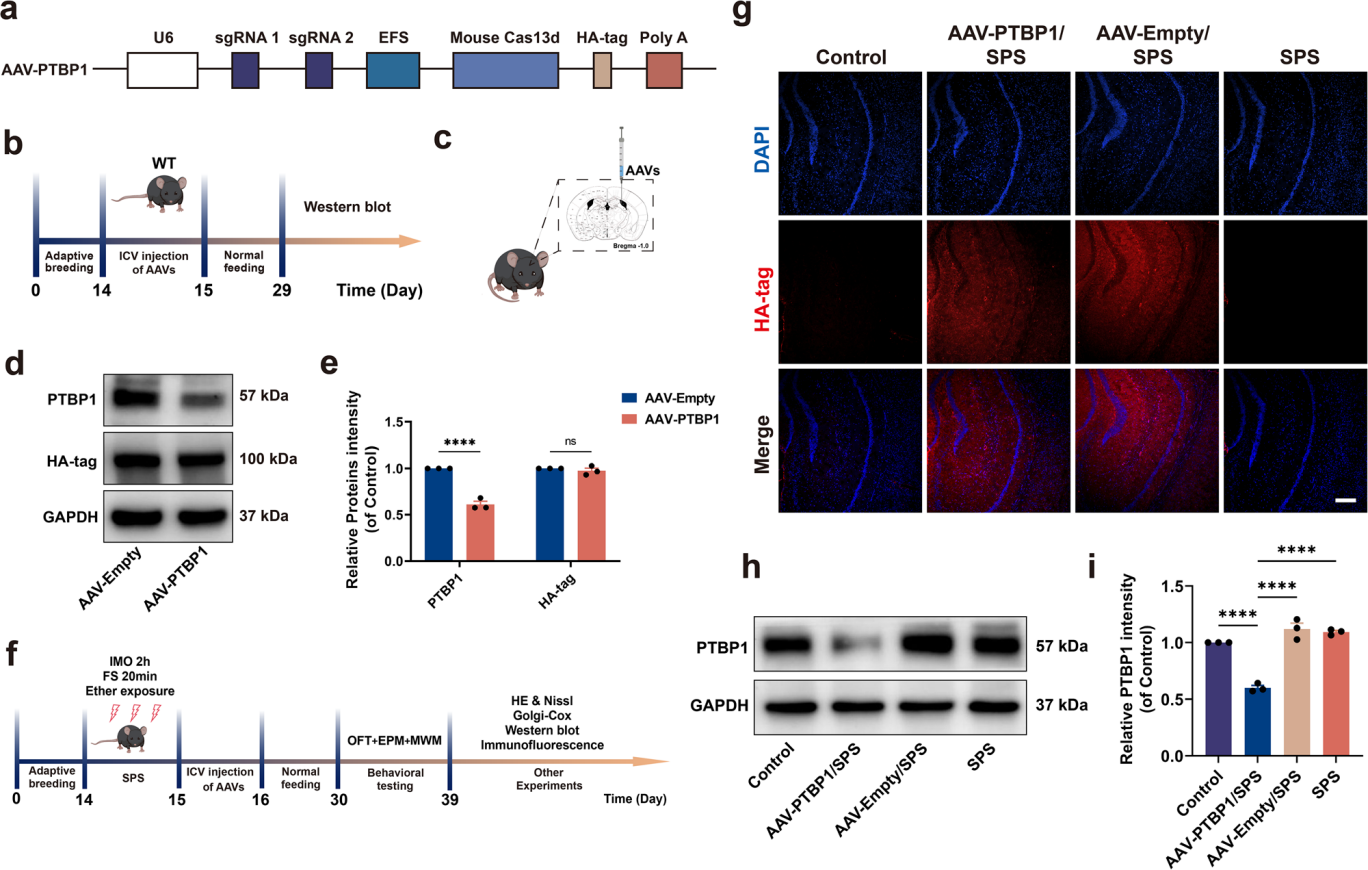
Experimental protocol and validation of PTBP1 KD. **a** The plasmid map of the AAV-PTBP1 KD virus (AAV-CasRx-PTBP1-HA). **b** The experimental protocol for in vivo validation of PTBP1 KD. **c** Schematic diagram of stereotaxic injection into the brain. **d** Validation of AAV-mediated PTBP1 KD in normal mice by western blot analysis of hippocampal PTBP1 and HA-tag. **e** Quantitative analysis of western blot results for PTBP1 and HA-tag in normal PTBP1 KD mice. **f** Experimental protocol to investigate the effects of intracorporeal PTBP1 KD in normal and PTSD mice. **g** Immunofluorescence staining for HA-tag (red) in normal and PTSD mice. Scale bar, 200 µm. **h** Validation of AAV-mediated PTBP1 KD in normal and PTSD mice by western blot analysis of hippocampal PTBP1. **i** Quantitative analysis of western blot results for PTBP1 in normal and PTSD mice. WT, wild type; IMO, immobilization on boards; FS, forced swimming. Data are expressed as mean ± s.e.m. (*n* = 3), ****P < 0.0001; ns, not significant.

To assess the KD efficiency of PTBP1 in vivo, the present study administered AAV-PTBP1-HA virus to the lateral ventricle of mice in the PTBP1 KD group (AAV-PTBP1) and AAV-Empty-HA virus to the control group (AAV-Empty). Two weeks post-injection, Western blot analysis was used to detect the expression of PTBP1 and HA-tag in the hippocampus (Fig. 1a–c). The results demonstrated that PTBP1 expression was significantly downregulated in the hippocampus, while no significant difference was observed in HA-tag expression. (Fig. 1d,e). Subsequently, to explore the effect and potential mechanism of downregulating hippocampal PTBP1 on the behavior of PTSD mice, AAV-PTBP1/SPS and AAV-Empty/SPS groups were injected into the lateral ventricle on the second day following modeling (Fig. 1f). After tissue collection, we examined the expression of HA-tag and PTBP1 to investigate the expression changes of PTBP1 under SPS-induced stress and to validate the KD efficiency of the AAV in the context of PTSD. The results showed that strong HA-tag signals were detected in the hippocampus of both the AAV-PTBP1/SPS and AAV-Empty/SPS groups, while no HA-tag signal was observed in the control and SPS groups that did not receive viral injections (Fig. 1g). Furthermore, PTBP1 expression in the AAV-PTBP1/SPS group was significantly lower than in the AAV-Empty/SPS group. Compared with the control group, both the AAV-Empty/SPS and SPS groups showed a slight upregulation of PTBP1 expression, but the difference was not statistically significant (Fig. 1h,i). These findings indicate that the AAV was successfully expressed under pathological conditions and effectively downregulated PTBP1, while PTBP1 expression itself did not change significantly under stress conditions alone.

### PTBP1 KD improves abnormal behaviors in PTSD mice

**Fig. 2:**
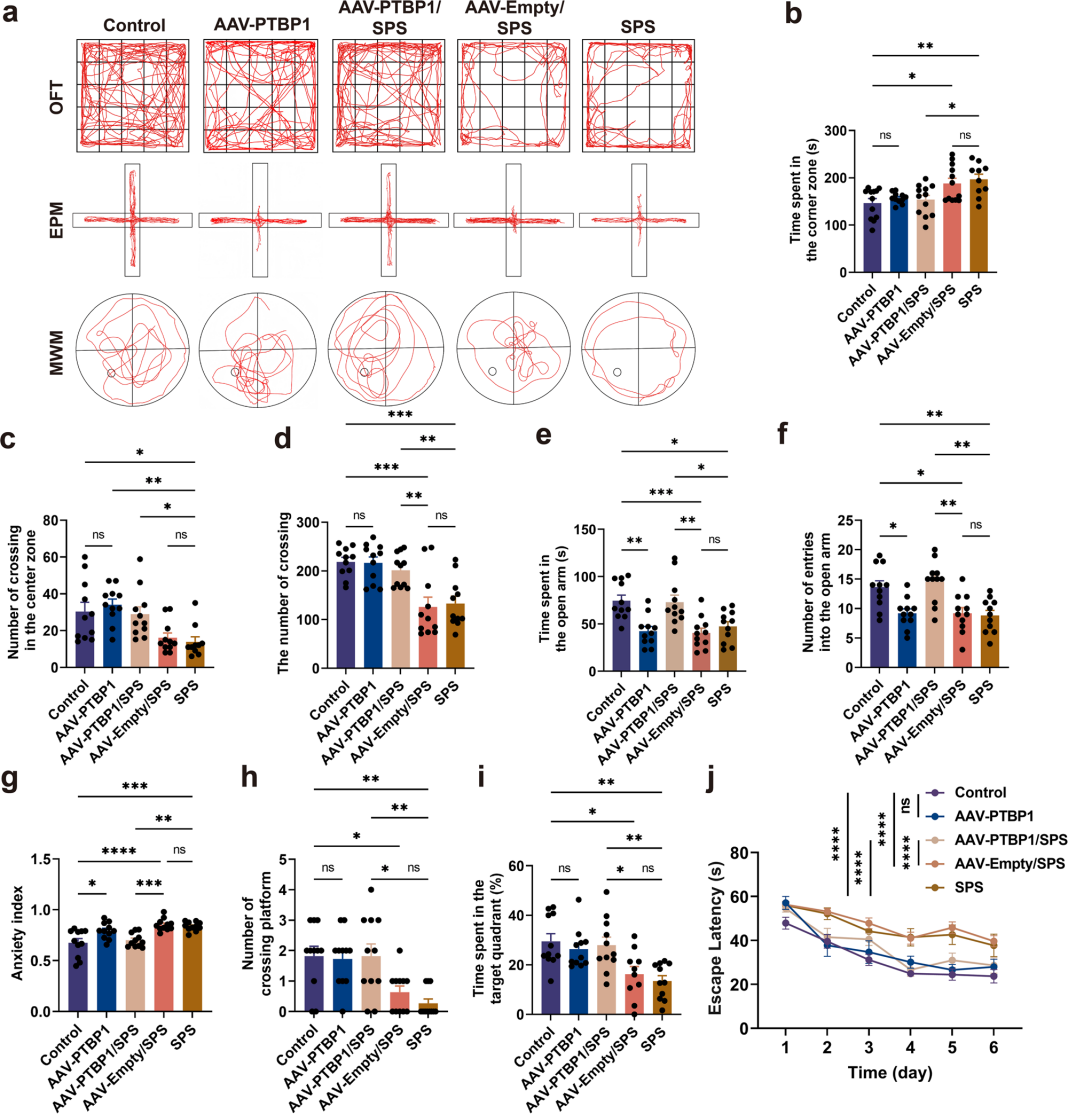
PTBP1 KD perturbs behavior in normal mice while improving behavioral deficits in PTSD mice. **a** The locomotor trajectories of mice within OFT, EPM and MWM. **b** Time spent in corner zone in the OFT. **c** The number of crossings in the center zone in the OFT. **d** The number of crossings in the OFT. **e** Time spent in open arm in the EPM. **f** The number of entries into open arm in the EPM. **g** Anxiety index in the EPM. **h** The number of crossing platforms in the MWM. **i** Proportion of time spent in the target quadrant in the MWM. **j** Escape latency in the MWM. In the EPM, the vertical arm is the open arm and the horizontal arm is the closed arm. Data are expressed as mean ± s.e.m. (*n* = 10–12), *P < 0.05, **P < 0.01, ***P < 0.001, ****P < 0.0001; ns, not significant.

Behavioral tests (OFT, EPM and MWM) were used to evaluate the behavioral performance of mice (Fig. 1f). The trajectories of the mice in the behavioral experiments are shown in Fig. 2a. The statistical analysis demonstrated significantly prolonged corner zone duration in SPS group mice versus controls during OFT, indicating the development of fear or depression-like emotions in the PTSD mice (Fig. 2b). In addition, the central and total crossing frequencies of the SPS group mice in the OFT were markedly decreased, without any physical damage, suggesting that SPS stimulation inhibited the mice’s locomotor interest (Fig. 2c,d). EPM testing revealed significantly reduced time spent in the open arm and decreased entries into the open arm in the SPS group, accompanied by a substantially elevated anxiety index, indicating fear and anxiety-like behaviors (Fig. 2e–g). MWM testing revealed spatial learning and memory deficits in SPS-model mice, indicated by reduced target quadrant occupancy, decreased platform crossings and prolonged escape latency (Fig. 2h–j). These abnormal behaviors were all significantly alleviated by PTBP1 KD intervention (Fig. 2a–j). PTBP1 KD treatment significantly alleviated the fear of PTSD mice in the OFT, improved their motor and exploration abilities, significantly reduced their anxiety in the EPM and enhanced their spatial learning and memory abilities in the MWM. In conclusion, PTBP1 KD effectively ameliorated abnormal behaviors in PTSD mice. It is noteworthy that, when examining the impact of PTBP1 KD on behavioral baseline in normal mice, we found that downregulation of PTBP1 did not significantly affect exploratory and locomotor activity in the OFT or spatial learning and memory in the MWM (Fig. 2a–d,h–j). However, in the EPM test, the mice exhibited significant anxiety-like behavior (Fig. 2e–g).

### PTBP1 KD rescued the abnormal loss of hippocampal neurons in PTSD mice by inhibiting apoptosis

**Fig. 3:**
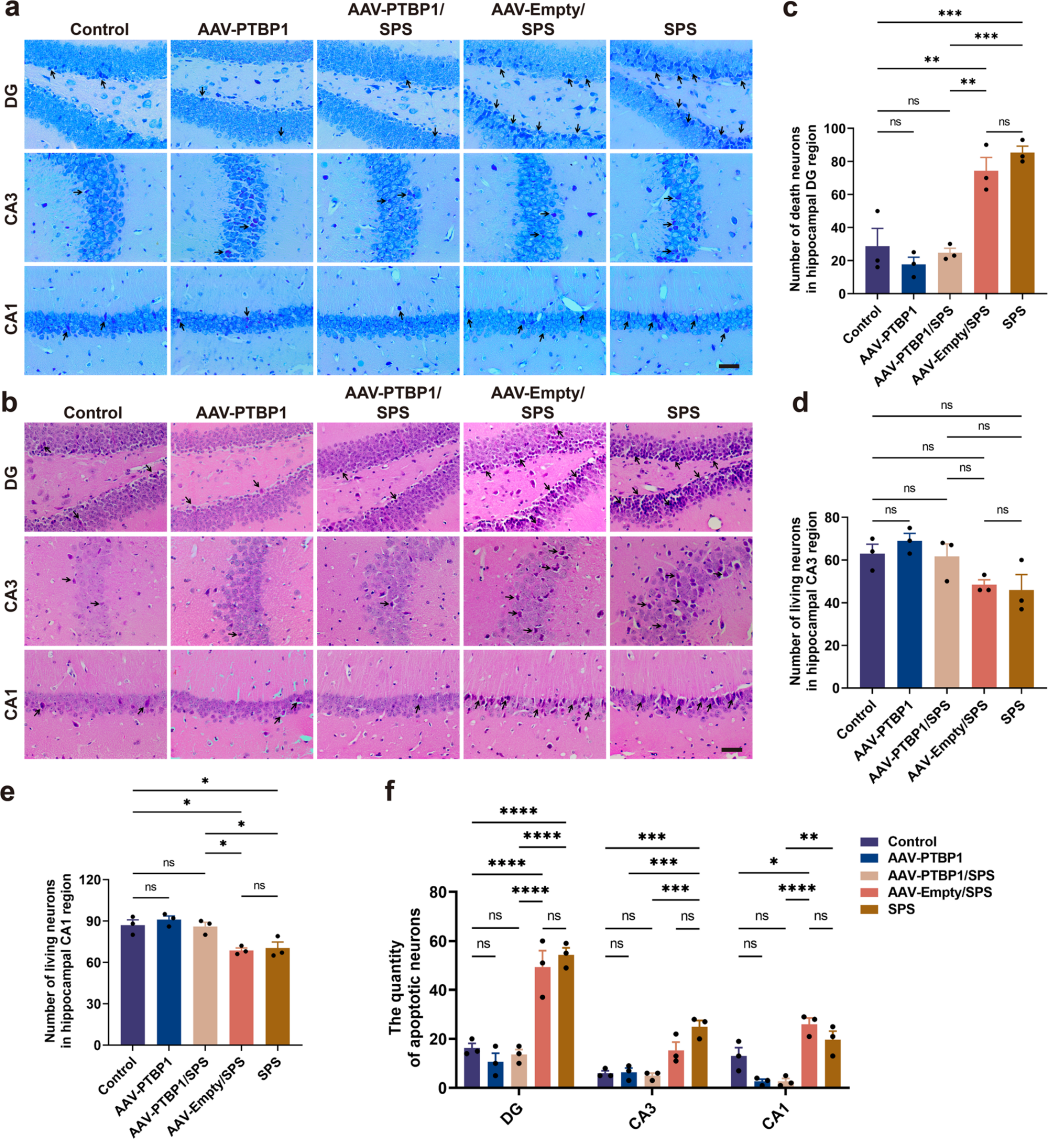
PTBP1 KD reduces hippocampal neuronal loss in SPS-induced PTSD mice. **a** Nissl staining of the hippocampus in mouse brain. The black arrow indicates damaged cells. Scale bar, 50 µm. **b** HE staining of the hippocampus in mouse brain. The black arrow indicates apoptotic-like cells. Scale bar, 50 µm. **c**–**e** Statistical results of the neuron death counts in the DG region (**c**), as well as the neuron survival count in the CA3 (**d**) and CA1 (**e**) regions. **f** Quantitative analysis of apoptotic-like cells in the DG, CA3 and CA1 subregions of the mouse hippocampus. Data are expressed as mean ± s.e.m. (*n* = 3), *P < 0.05, **P < 0.01, ***P < 0.001, ****P < 0.0001; ns, not significant.

To investigate whether PTBP1 KD-mediated behavioral improvements in PTSD mice are associated with hippocampal neuronal loss, we examined the correlation between PTBP1 KD and hippocampal neuronal loss, concurrently exploring underlying mechanisms. Nissl staining and HE staining were used to detect the loss and survival of hippocampal neurons at the tissue level. Nissl staining revealed numerous intensely stained nuclei in hippocampal dentate gyrus (DG) and cornu ammonis 1 (CA1) subregions of both SPS and AAV-Empty/SPS groups, while quantitative analysis demonstrated significantly reduced surviving neuron counts in these regions. (Fig. 3a,c,e). In the CA3 subregion, neuronal loss was observed in both the SPS and AAV-Empty/SPS groups, whereas the number of surviving neurons did not differ significantly among the groups (Fig. 3a,d). After PTBP1 KD intervention, there was a significant increase in the number of surviving neurons in the hippocampal DG and CA1 subregions compared with the SPS group (Fig. 3a,c,e). HE staining revealed a significantly greater number of pathologically altered neurons in the hippocampus of PTSD model mice compared with the control group (Fig. 3b). Meanwhile, the number of injured cells decreased in the AAV-PTBP1/SPS group. Notably, there was a significant reduction in apoptosis-like cells, suggesting that PTBP1 alleviates SPS-induced damage to the hippocampus (Fig. 3b,f). Furthermore, Nissl and HE staining revealed that, compared with the control group, PTBP1 KD did not induce significant pathological damage and neuron loss in the hippocampus of normal mice, suggesting that other potential factors may mediate the anxiety-like behavior caused by PTBP1 downregulation in normal mice (Fig. 3a–f).

**Fig. 4:**
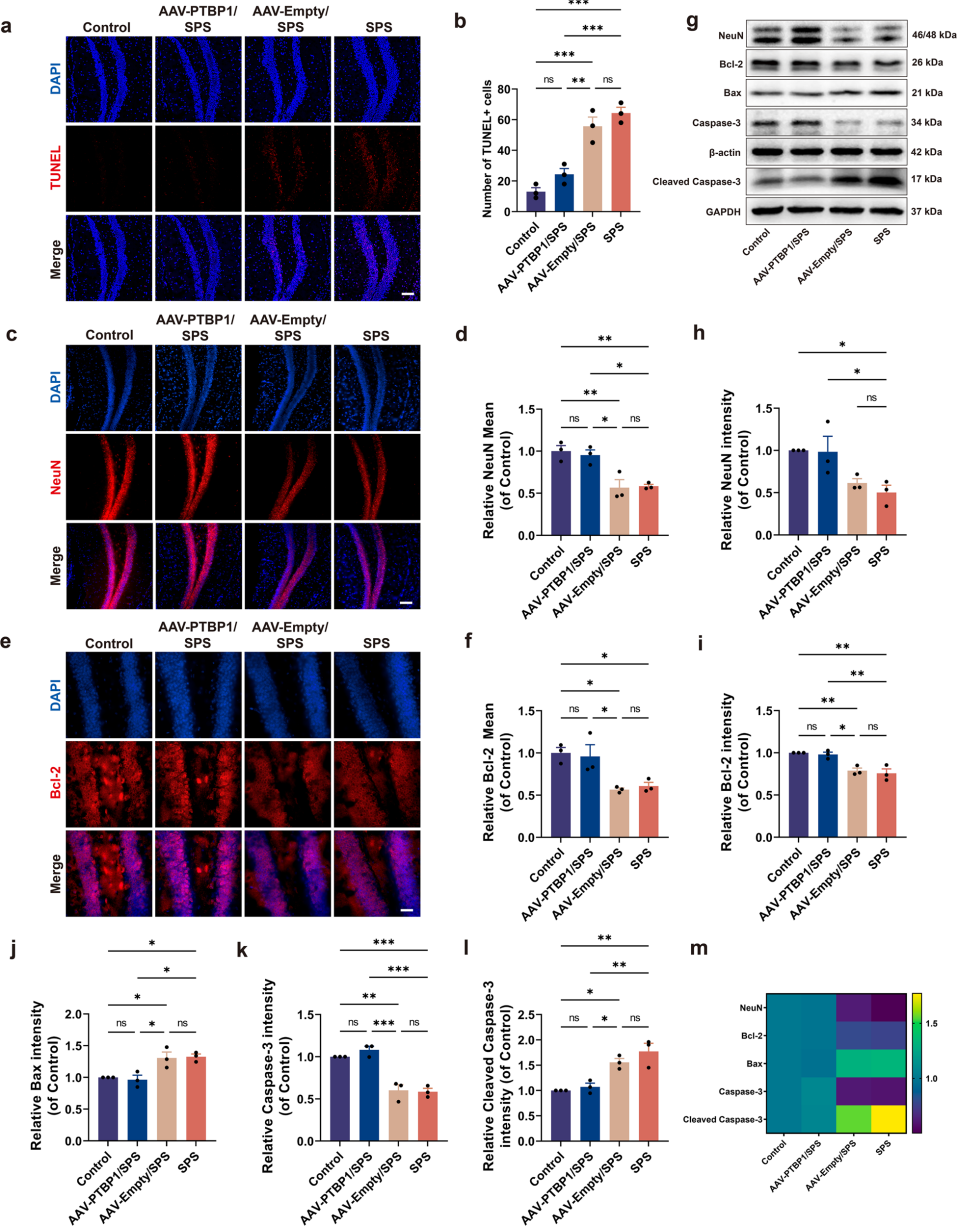
PTBP1 KD rescues hippocampal neuron loss by inhibiting apoptosis. **a** TUNEL staining of the hippocampus in mouse brain. Scale bar, 100 µm. **b** Quantitative analysis of TUNEL-positive cells in the DG region. **c** Immunofluorescence staining for NeuN (red). Scale bar, 100 µm. **d** Statistical analysis of mean fluorescence intensity of NeuN. **e** Immunofluorescence staining for Bcl-2 (red). Scale bar, 50 µm. **f** Statistical analysis of mean fluorescence intensity of Bcl-2. **g** Western blot analysis of NeuN and apoptosis-related proteins, including Bcl-2, Bax, Caspase-3 and Cleaved Caspase-3. **h**–**l** Quantitative analysis of western blot results for NeuN (**h**), Bcl-2 (**i**), Bax (**j**), Caspase-3 (**k**) and Cleaved Caspase-3 (**l**). **m** Heatmap representation of western blot experimental results. Data are expressed as mean ± s.e.m. (*n* = 3), *P < 0.05, **P < 0.01, ***P < 0.001; ns, not significant.

Using TUNEL-based apoptosis detection, we observed notably fewer TUNEL-positive cells following PTBP1 suppression (Fig. 4a,b). In addition, we examined NeuN expression, a mature neuronal marker, in the hippocampus using immunofluorescence and western blot analyses. The expression of NeuN decreased significantly after SPS stimulation, while it was increased in the AAV-PTBP1/SPS group. These findings demonstrated that PTBP1 KD effectively rescued the abnormal loss of hippocampal neurons in PTSD mice (Fig. 4c,d,g,h,m).

Next, western blot analysis was performed to quantify the protein levels of apoptosis-related proteins Bcl-2, Bax, Caspase-3 and Cleaved Caspase-3. The results illustrated that the expression of anti-apoptotic factors Bcl-2 and Caspase-3 was downregulated, and the expression of the pro-apoptotic factor Bax and Cleaved caspase-3 was upregulated in the SPS group. By contrast, the AAV-PTBP1/SPS group demonstrated the opposite trend (Fig. 4g,i–m). The changes in Bcl-2 expression were also confirmed by immunofluorescence staining (Fig. 4e,f). The above results indicate that PTBP1 KD mitigated the SPS-induced hippocampal neurons loss by inhibiting apoptosis.

### PTBP1 KD partially promotes synaptic plasticity in hippocampal neurons of PTSD mice

**Fig. 5:**
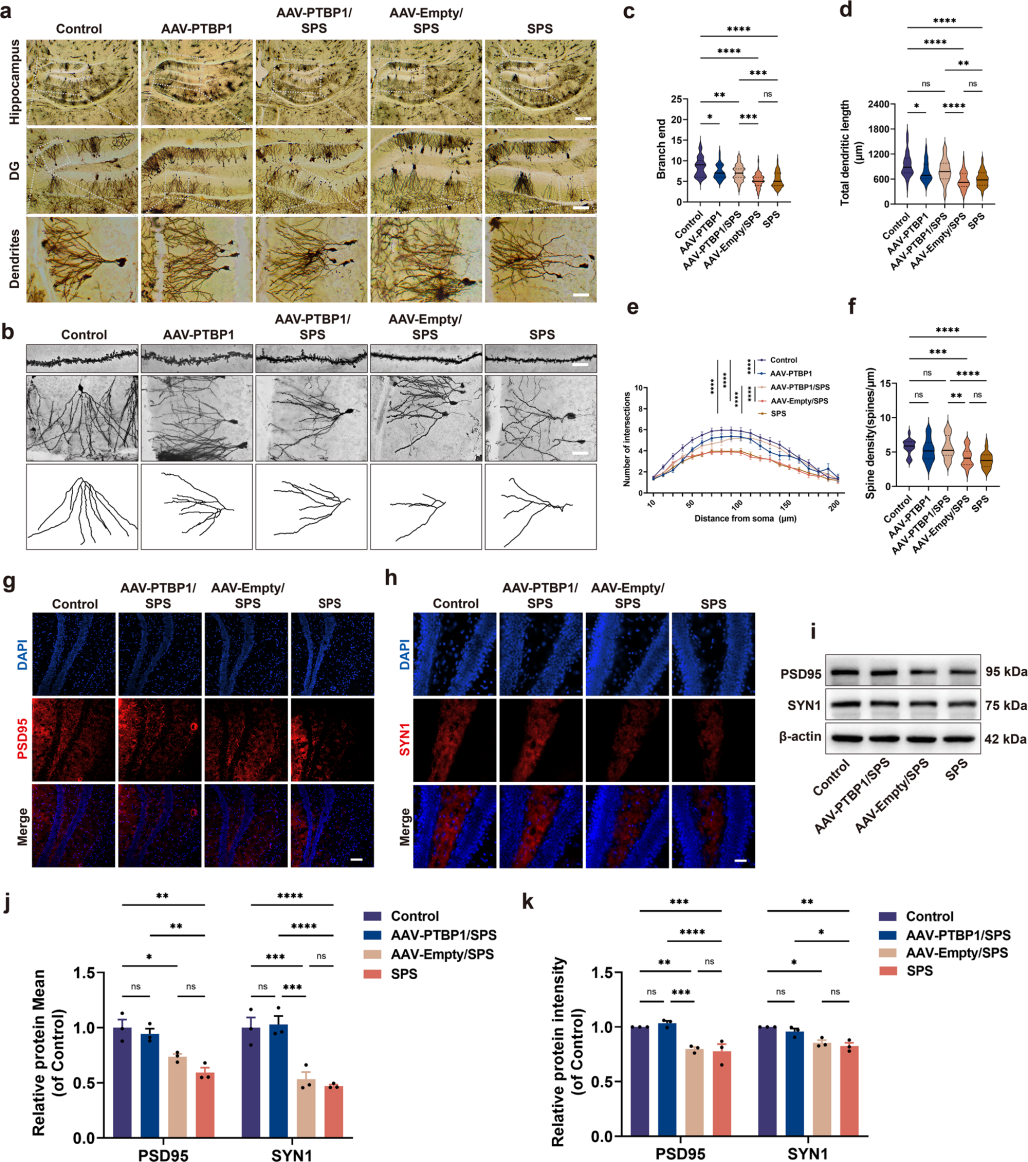
The effect of PTBP1 KD on synaptic plasticity in hippocampal neurons of normal and PTSD mice. **a** Golgi-Cox staining. Scale bars: hippocampus, 500 µm; DG, 200 µm; dendrites, 50 µm. **b** Dendritic spine (top), single neuron (middle) and its corresponding morphological simulation image (bottom). Scale bars: top, 5 µm; middle, 50 µm. **c** The number of terminal branches on dendrites. **d** Total dendritic length. **e** Sholl analysis. **f** Dendritic spine density. **g** Immunofluorescence staining of PSD95 (red) in the DG region of the hippocampus. Scale bar, 100 µm. **h** Immunofluorescence staining of SYN1 (red) in DG region of the hippocampus. Scale bar, 50 µm. **i** Western blot analysis of PSD95 and SYN1. **j** Statistical analysis of the mean fluorescence intensity of PSD95 and SYN1. **k** Quantitative analysis of western blot results for PSD95 and SYN1. Data are expressed as mean ± s.e.m. (**c**–**f**: *n* = 3, conducted a statistical analysis on 10–15 neurons per sample; **j** and **k**: *n* = 3), *P < 0.05, **P < 0.01, ***P < 0.001. ****P < 0.0001; ns, not significant.

Hippocampal information processing efficacy is contingent upon synaptic adaptability, which requires precise regulation of dendritic architecture and spine developmental dynamics. In this study, Golgi-Cox staining was used to observe synaptic plasticity in hippocampal neurons. The results revealed that hippocampal neuronal dendrites in the SPS group were shorter and arranged in a disordered manner, particularly in neurons of DG region of the hippocampus, confirming that SPS stimulation led to abnormal dendrite growth in hippocampal neurons of PTSD-like mice. By contrast, the AAV-PTBP1/SPS group exhibited improved dendritic growth status (Fig. 5a). Furthermore, hippocampal dendritic characteristics were systematically characterized through quantitative length measurement, branch terminal counting and computational Sholl analysis for complexity determination. Quantitative analysis revealed significant reductions in total dendrite length, the number of dendrite terminal branches and dendrite complexity in the SPS group, with improvements after PTBP1 KD (Fig. 5b–e). Notably, although PTBP1 KD partially ameliorated dendritic growth, it remained significantly lower than in controls, indicating that dendrite development was still suppressed (Fig. 5c–e). By examining dendritic spine density, it was found that spine density decreased after SPS stimulation, while it significantly increased in the AAV-PTBP1/SPS group, indicating that dendrite spines were damaged in the SPS group, whereas PTBP1 KD facilitated dendrite spine growth (Fig. 5b,f). In addition, combined western blot and immunofluorescence analyses were performed to quantify the expression of PSD95 and Synapsin I (SYN1). Consistent with the dendrite spine density results, PSD95 and SYN1 expression significantly decreased after SPS stimulation but were significantly upregulated after PTBP1 KD (Fig. 5g–k). These findings suggested that knocking down PTBP1 can enhance synaptic plasticity in hippocampal neurons of PTSD-like mice to some extent by promoting PSD95 and SYN1 expression and increasing dendrite spine density. However, it seems to simultaneously activate certain adverse factors that inhibit dendrite growth itself, limiting its effectiveness in promoting hippocampal synaptic plasticity.

Similarly, analogous phenomena were observed in the AAV-PTBP1 group. Compared with the control group, although the AAV-PTBP1 group exhibited no signs of disorganized dendritic branching patterns nor significant differences in dendritic spine density, examinations revealed statistically significant reductions in total dendritic length, number of dendritic terminal branches and dendritic complexity (Fig. 5a–f). These findings indicate that PTBP1 downregulation may potentially exert detrimental effects on synaptic plasticity. In summary, PTBP1 KD only partially promotes synaptic plasticity in hippocampal neurons.

### PTBP1 KD inhibits axon regeneration by activating the Nogo-A/NgR/RhoA/ROCK2 pathway in hippocampal neurons of PTSD mice

**Fig. 6:**
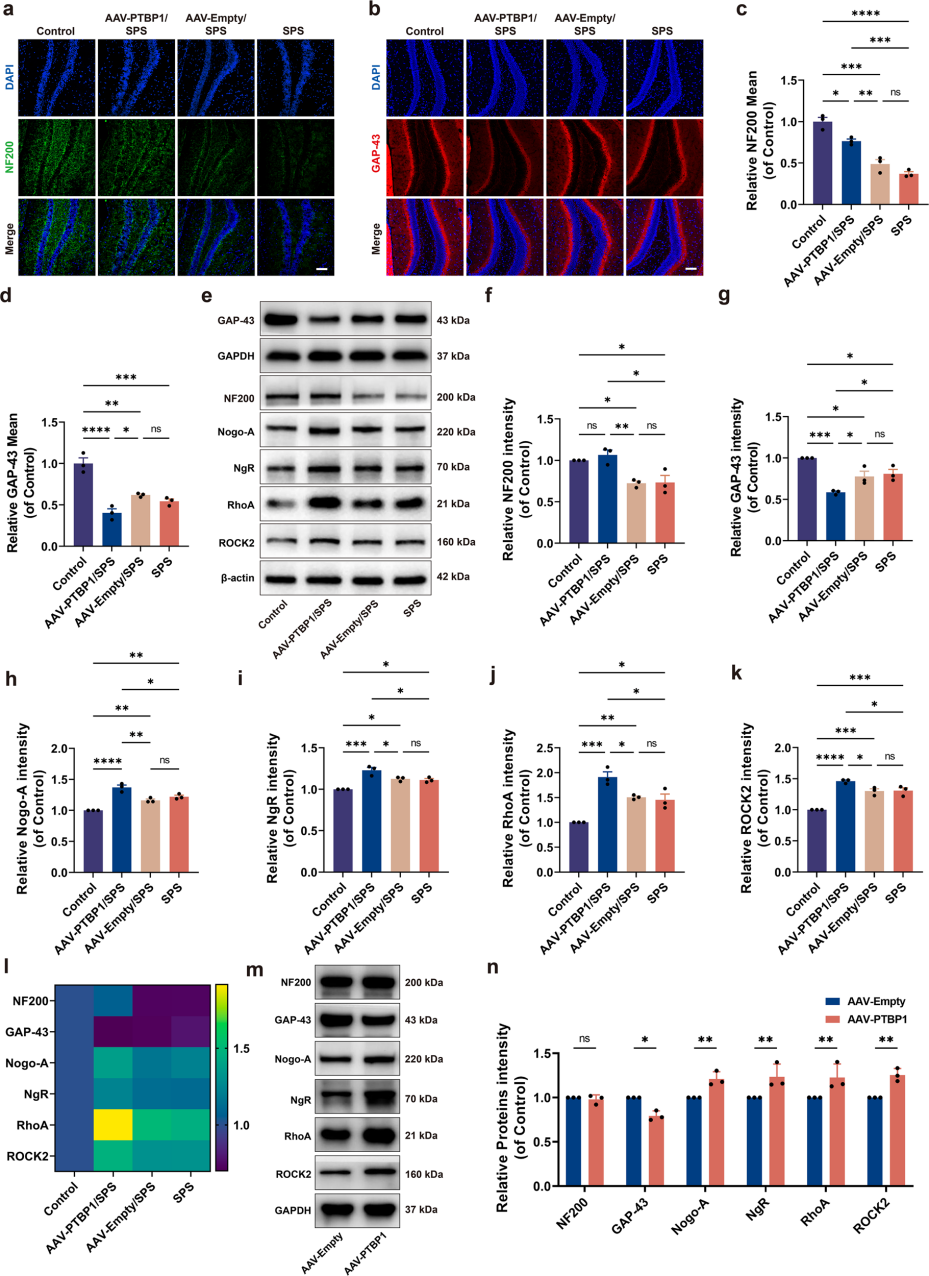
Regulation of axonal regeneration by PTBP1 KD via the Nogo-A/NgR/RhoA/ROCK2 axis in normal and PTSD mice. **a**, **b** Immunofluorescence staining of NF200 (green) (**a**) and GAP-43 (red) (**b**) in the DG region of the hippocampus. Scale bar, 100 µm. **c**, **d** Statistical analysis of the average fluorescence intensity of NF200 (**c**) and GAP-43 (**d**). **e** Western blot analysis of NF200, GAP-43, Nogo-A, NgR, RhoA and ROCK2. **f**–**k** Quantitative analysis of western blot results for NF200 (**f**), GAP-43 (**g**), Nogo-A (**h**), NgR (**i**), RhoA (**j**) and ROCK2 (**k**). **l** The heatmap representation of western blot results. **m** Western blot analysis of PTBP1 KD-induced changes in the Nogo-A/NgR/RhoA/ROCK2 pathway and NF200/GAP-43 in normal mice. **n** Quantitative analysis of western blot results for the Nogo-A/NgR/RhoA/ROCK2 pathway and NF200/GAP-43 in normal mice. Data are expressed as mean ± s.e.m. (*n* = 3), *P < 0.05, **P < 0.01, ***P < 0.001. ****P < 0.0001; ns, not significant.

Structural stabilization and re-extension of neuronal axons are crucial for their regenerative capacity. Neurofilament 200 (NF200) constitutes a major structural element of neuronal cytoskeletons, predominantly localized to distal axonal segments where it preserves structural integrity. Growth-associated protein-43 (GAP-43) is primarily situated in neuronal growth cones, playing an essential role in axonal elongation through dynamic cytoskeletal remodeling and morphological adaptation. To assess PTBP1 KD effects on hippocampal axonal regeneration in PTSD mice, we used immunofluorescence and western blot analyses to quantify established biomarkers NF200 and GAP-43. Western blot analysis revealed that both markers were significantly downregulated in the SPS group. Interestingly, PTBP1 KD selectively restored NF200 levels, while GAP-43 expression instead exhibited a significant decrease (Fig. 6e–g,l). Simultaneously, immunofluorescence analysis was performed for NF200 and GAP-43 in the hippocampal DG region. The results demonstrated that, compared with the AAV-Empty/SPS group, the AAV-PTBP1/SPS group exhibited a partial upregulation of NF200 expression, although it remained significantly lower than that in the normal group. Conversely, GAP-43 expression was significantly downregulated compared with the AAV-Empty/SPS group, which was consistent with the western blot results (Fig. 6a–d).

Here, there seems to be a mechanism limiting the recovery of axonal regenerative capacity by PTBP1 KD, particularly for axonal elongation. Meanwhile, based on the aforementioned adverse effects of PTBP1 KD on synaptic plasticity, we focused on the Nogo-A/NgR/RhoA/ROCK2 axis, which is highly relevant to synaptic plasticity and axonal regeneration. Nogo-A expression demonstrated marked elevation in the SPS group versus the control group, with the AAV-PTBP1/SPS group displaying analogous upregulation relative to the SPS group (Fig. 6e,h,l). It is well known that Nogo-A-mediated activation of NgR has detrimental effects on synaptic plasticity and axonal regeneration. Consistent with our expectations, activation of the Nogo-A/NgR/RhoA/ROCK2 signaling pathway was detected at the protein level in both the SPS and AAV-Empty/SPS groups. Moreover, significantly elevated activation levels were observed in the AAV-PTBP1/SPS group when compared with both the SPS and AAV-Empty/SPS groups (Fig. 6e,h–l). This finding implies that PTBP1 KD may exacerbate the inhibition of dendrite growth and axonal regeneration by enhancing the activation of this inhibitory signal for nerve regeneration. To further elucidate whether the axonal regeneration impairment caused by PTBP1 KD-mediated through Nogo-A and its downstream signaling activation represents a pathology-specific consequence or reflects a general function of PTBP1, we knocked down PTBP1 in normal mice to examine alterations in this pathway and its downstream effects. The results demonstrated that PTBP1 KD alone was sufficient to activate the Nogo-A/NgR/RhoA/ROCK2 signaling pathway and led to the downstream GAP-43 expression downregulation (Fig. 6m,n). These findings collectively indicate that PTBP1 downregulation impairs both dendritic growth and axonal regeneration by activating the Nogo-A/NgR/RhoA/ROCK2 axis, representing a general mechanism of PTBP1-mediated regulation.

### NgR KD suppresses activation of the RhoA/ROCK2 pathway, reversing dendritic growth abnormalities and axonal regeneration impairments induced by SPS stimulation and PTBP1 KD

**Fig. 7:**
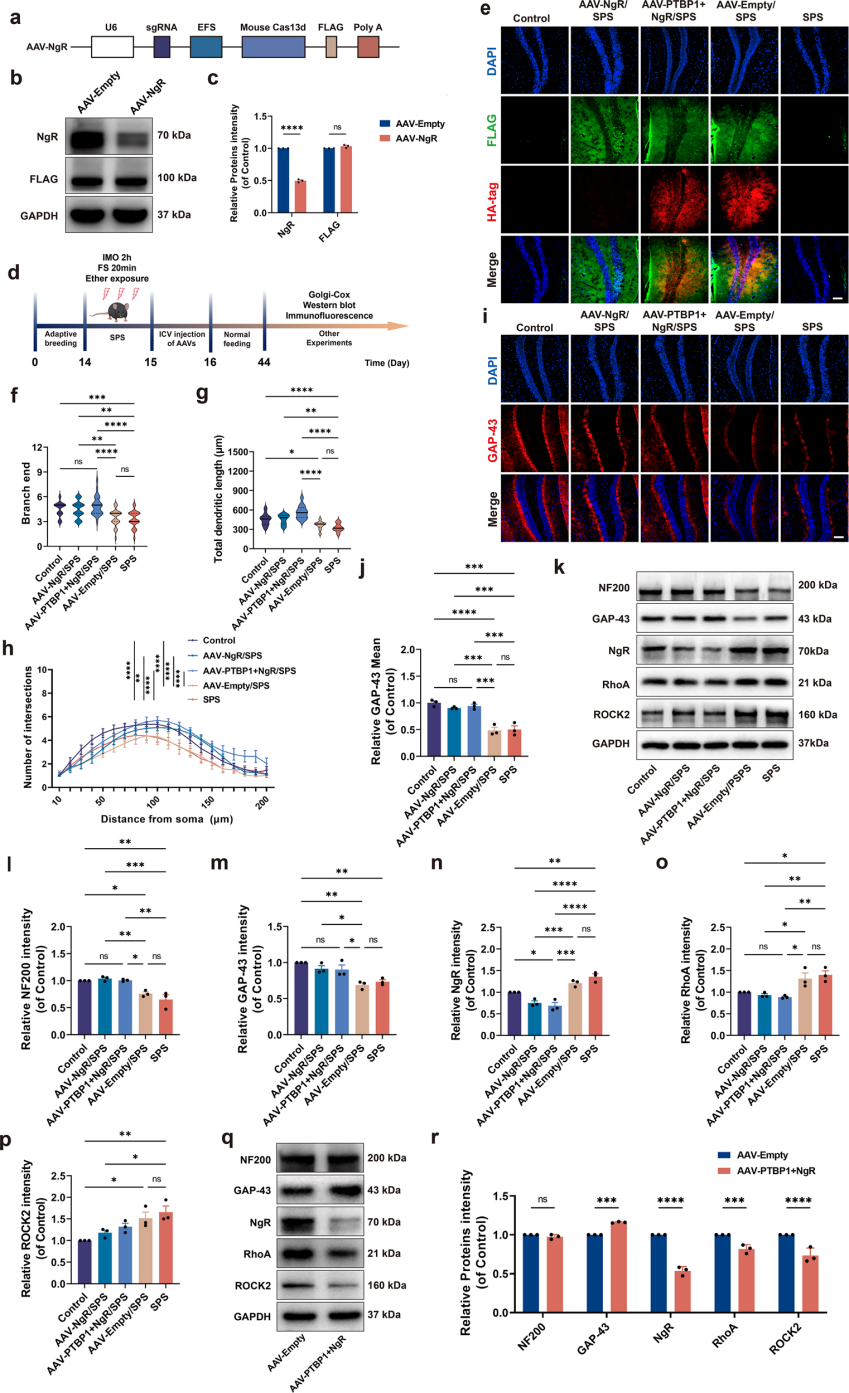
NgR KD reverses dendrite abnormalities and axonal regeneration impairments induced by SPS and PTBP1 KD. **a** The plasmid map of the AAV-NgR KD virus (AAV-CasRx-NgR-FLAG). **b** Utilization of western blot analysis to detect NgR and FLAG expression in the hippocampus of normal mice. **c** Quantitative analysis of western blot results for NgR and FLAG in the hippocampus of normal mice. **d** Experimental protocol for investigating the effect of combined KD of PTBP1 and NgR on axon regeneration in normal and PTSD mice. **e** Immunofluorescence detection of HA-tag (red) and FLAG tag (green) proteins. Scale bar, 100 µm. **f** The number of terminal branches on dendrites. **g** Total dendritic length. **h** Sholl analysis. **i** Immunofluorescence staining of GAP-43 (red) in the DG region of the hippocampus. Scale bar, 100 µm. **j** Statistical analysis of the average fluorescence intensity of GAP-43. **k** Western blot analysis of NF200, GAP-43, NgR, RhoA and ROCK2. **l**–**p** Quantitative analysis of western blot results for NF200 (**l**), GAP-43 (**m**), NgR (**n**), RhoA (**o**) and ROCK2 (**p**). **q** Western blot analysis of PTBP1 KD-induced changes in the NgR/RhoA/ROCK2 pathway and NF200/GAP-43 in normal mice. **r** Quantitative analysis of western blot results for the NgR/RhoA/ROCK2 pathway and NF200/GAP-43 in normal mice. Data are expressed as mean ± s.e.m. (**f**–**h**: *n* = 3, conducted a statistical analysis on 10–15 neurons per sample; **c**, **j**, **l**–**p** and **r**: *n* = 3), *P < 0.05, **P < 0.01, ***P < 0.001. ****P < 0.0001; ns, not significant.

Based on the aforementioned results, we attempted to target the Nogo-A downstream receptor NgR by lateral ventricle injection of AAV-NgR-FLAG. This intervention significantly downregulated NgR protein expression in the AAV-NgR group mice (Fig. 7a–c). Through NgR KD-mediated inhibition of downstream signaling, we systematically evaluated the therapeutic effects of NgR KD on dendritic arborization and axonal regrowth in PTSD mice (Fig. 7d).

Immunofluorescence staining detected HA-tag and FLAG expressions. FLAG was exclusively expressed in the AAV-NgR/SPS group, while both tags were present in AAV-PTBP1+NgR/SPS and AAV-Empty/SPS groups, confirming viral delivery efficacy (Fig. 7e). Western blot analysis of NgR protein expression further confirmed the successful KD of NgR (Fig. 7k,n). Consistent with the expected results, Golgi-Cox staining illustrated that, after NgR KD or PTBP1 and NgR KD in PTSD mice, their dendrite branching number and dendrite length were significantly restored. Sholl analysis results indicated a significant increase in dendrite complexity in the KD groups, suggesting that NgR KD alone restored dendrite growth to normal levels. The results for PTBP1 and NgR KD were consistent with those for NgR KD, indicating that PTBP1 and NgR KD reversed the inhibition of dendrite growth caused by PTBP1 KD (Fig. 7f–h). Western blot and immunofluorescence analyses showed that NF200 and GAP-43 expression in the AAV-NgR/SPS group was upregulated to control levels, indicating NgR KD restored axon regeneration (Fig. 7i–m). Combined PTBP1 and NgR downregulation alleviated PTBP1 KD-induced axonal regeneration suppression (Fig. 7i–m). We further examined the expression of the downstream signaling molecules RhoA and ROCK2 of NgR. Downstream RhoA and ROCK2 expressions were significantly inhibited in AAV-NgR/SPS and AAV-PTBP1+NgR/SPS groups versus SPS group (Fig. 7k,n–p), confirming that NgR KD counteracts SPS and PTBP1 KD-driven RhoA/ROCK2 activation. Similarly, we knocked down both PTBP1 and NgR in normal mice and examined the NgR/RhoA/ROCK2 signaling pathway along with NF200/GAP-43 expression. The results demonstrated that concomitant NgR KD rescued the axonal regeneration impairment induced by PTBP1 KD alone in normal mice (Fig. 7q,r).

The above results further confirm that activation of the Nogo-A/NgR/RhoA/ROCK2 axis underlies both dendritic damage and deficits in axonal regeneration in PTBP1-KD PTSD mice.

### PTBP1 and NgR KD demonstrates greater potential benefits for PTSD-like behaviors over PTBP1 KD alone, while also rescuing anxiety in normal PTBP1-KD mice

**Fig. 8:**
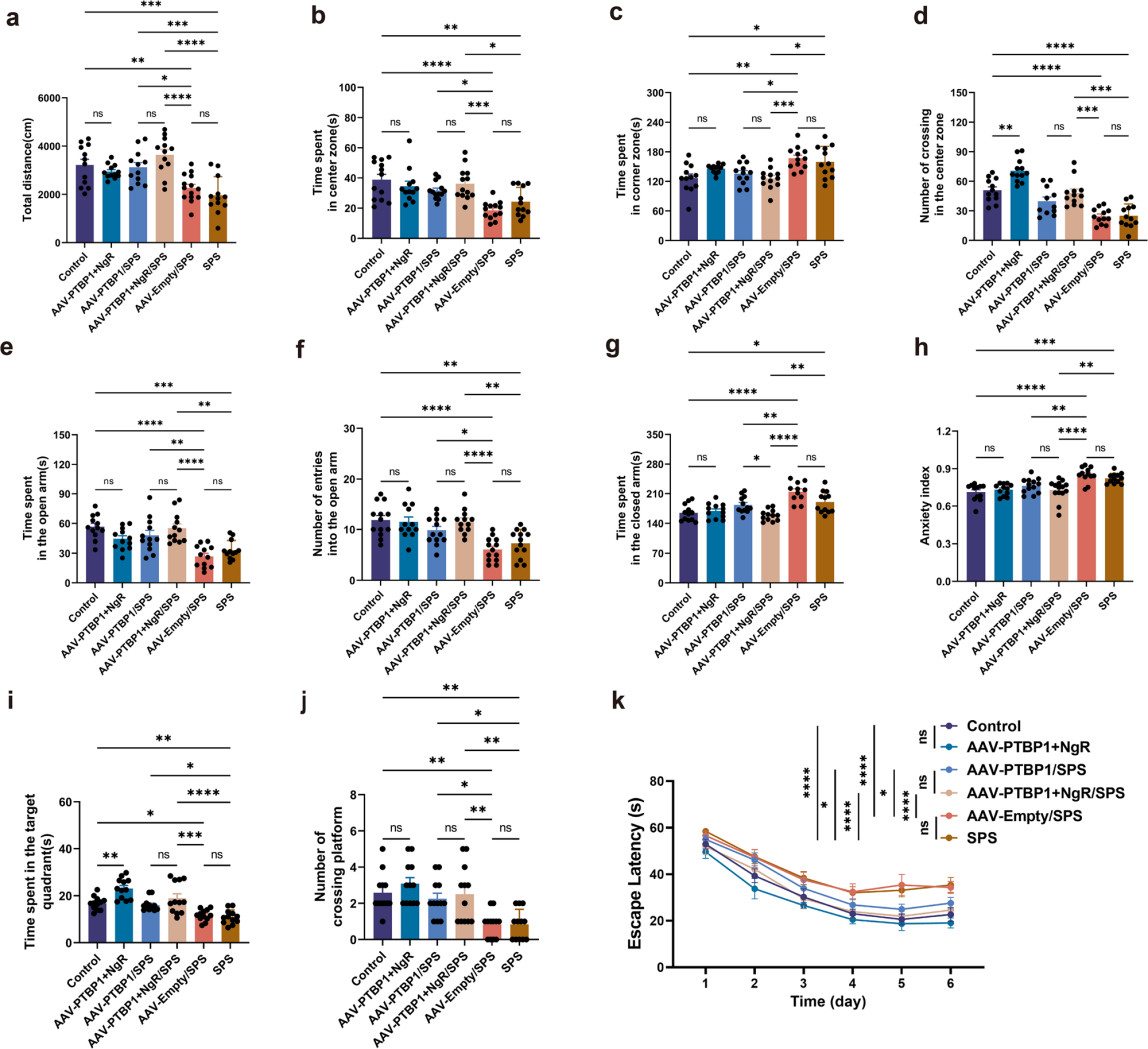
PTBP1 and NgR KD demonstrates a more potential benefit for PTSD-like behaviors over PTBP1 KD alone, while also rescuing anxiety in normal PTBP1-KD mice. **a** Total distance in OFT. **b** Time spent in center zone in the OFT. **c** Time spent in corner zone in the OFT. **d** The number of times the central zone was crossed in the OFT. **e** Time spent in open arm in the EPM. **f** The number of entries into the open arm in the EPM. **g** Time spent in closed arm in the EPM. **h** Anxiety index in the EPM. **i** Time spent in the target quadrant in the MWM. **j** The number of crossing platforms in the MWM. **k** Escape latency in the MWM. Data are expressed as mean ± s.e.m. (*n* = 11–13), *P < 0.05, **P < 0.01, ***P < 0.001. ****P < 0.0001; ns, not significant.

Finally, behavioral tests were conducted to determine whether combined KD of PTBP1 and NgR elicits superior behavioral effects compared with PTBP1 KD alone in both normal and PTSD mice. The OFT results demonstrated that, compared with the SPS group and AAV-Empty group, the AAV-PTBP1+NgR/SPS group had significantly increased total distance, time spent in the central zone and number of crossings in the center zone, while time spent in the corner zone was significantly lower (Fig. 8a–d). EPM results showed that, in the AAV-PTBP1+NgR/SPS group, both time spent in the open arm and the number of entries into the open arm significantly surpassed those in the SPS group and AAV-Empty group. Concurrently, time spent in the closed arm and the anxiety index was significantly reduced compared with both the SPS and AAV-Empty/SPS groups (Fig. 8e–h). MWM results revealed that, compared with the SPS group and AAV-Empty group, the AAV-PTBP1+NgR/SPS group significantly increased time spent in the target quadrant and the number of crossing platform, and significantly reduced the escape latency (Fig. 8i–k). Although the PTBP1 and NgR KD group showed significant improvement only in the time spent in the closed arms during the EPM test compared with the AAV-PTBP1/SPS group, its behavioral improvements relative to the AAV-PTBP1/SPS group demonstrated stronger statistical significance across various behavioral assays when compared with both the SPS group and the AAV-Empty/SPS group. In normal mice, combined KD of PTBP1 and NgR produced significant phenotypic reversals. Our previous findings demonstrated that PTBP1 KD alone could induce anxiety-like behavior in the EPM (Fig. 2a,e–g), whereas the current study reveals that co-KD of PTBP1 and NgR completely abolished this abnormal behavior (Fig. 8e–h). Furthermore, in the OFT, the AAV-PTBP1 group exhibited a significant increase the number of crossings in the center zone compared with the control group. Similarly, in the MWM, the time spent in the target quadrant was significantly prolonged in the AAV-PTBP1 group relative to controls (Fig. 8d,i).

In summary, these results demonstrate that, although the combined KD of PTBP1 and NgR did not demonstrate a more significant effect in alleviating PTSD-like behaviors compared with PTBP1 KD alone, its role in reducing both the molecular side effects and additional behavioral risks associated with the latter is advantageous. Mechanistically, NgR KD probably amplifies the beneficial effects of PTBP1 KD by blocking the activation of the Nogo-A/NgR/RhoA/ROCK2 signaling pathway.

## Discussion

The transdifferentiation of glial cells in situ through PTBP1 KD has been questioned by multiple laboratories. Some of these teams, despite improving disease phenotypes through PTBP1 KD, have not attributed this effect to transdifferentiation, and the underlying mechanisms remain unclear^18,38–40^. Interestingly, among the academic criticisms regarding the successful amelioration of disease phenotypes through PTBP1 KD via AAVs, nearly all have emphasized the issue of neuronal leakage^38–40^. Xie and colleagues demonstrated gene-dependent neuronal leakage from AAVs, and although PTBP1-dependent neuronal leakage has not been demonstrated, the existing results also illustrate the prevalence of the neuronal leakage problem from AAVs^38–41^. This implies that the use of AAVs as a gene delivery tool may lead to downregulation of PTBP1 expression in neurons. Given PTBP1’s extensive regulatory functions in neuronal development, elucidating the specific pathways through which its downregulation ameliorates disease phenotypes in neurons becomes a compelling research direction.

This study pioneered AAV-mediated PTBP1 targeting in PTSD treatment, contrasting with prior investigations that examined cell-type-specific KD effects, by instead assessing global hippocampal PTBP1 KD therapeutic impact on adult PTSD mice^17,42^. Consistent with prior research demonstrating behavioral normalization following PTBP1 downregulation in central nervous system (CNS) dysfunction models, our study achieved notable amelioration of fear, anxiety and spatial learning/memory deficits in PTSD mice through AAV-mediated PTBP1 KD. However, it is noteworthy that behavioral results indicate that PTBP1 KD alone may induce anxiety-like behavior in normal mice. By contrast, under PTSD-like pathological conditions, downregulation of PTBP1 conversely ameliorated anxiety-like behavior. These results suggest that the regulatory role of PTBP1 in anxiety-like behavior may be context dependent, indicating that intervention strategies solely targeting PTBP1 KD may carry potential risks. Studying the underlying mechanisms behind this phenomenon is an interesting task.

SPS exposure induces pathological neuronal loss in the hippocampal DG, CA1 and CA3 subfields, a phenomenon consistently reported in prior investigations^6,43^. PTBP1 KD effectively alleviated neuronal loss, consistent with prior evidence demonstrating its regulatory role in apoptosis via post-transcriptional alternative splicing of Bcl-2 family genes. Mechanistically, PTBP1 represses exon 3 inclusion of BCL-2-like 11 (BIM), a pro-apoptotic factor implicated in neurodegeneration, thereby enhancing BIM expression. Inverse regulation was observed for PTBP1 downregulation, which suppressed BIM-mediated apoptotic pathways^44^. Lin et al. established that PTBP1 KD obviously attenuates ceramidin-induced apoptosis through suppression of Bak1 and Cleaved Caspase-3 expression^22^. This mechanistic axis aligns with our current observations in PTSD mice, where PTBP1 KD similarly inhibited hippocampal apoptosis while specifically repressing Caspase-3 activation. Furthermore, systematic evaluation of Bcl-2 family dynamics (Bcl-2/Bax balance) and caspase activation profiles confirmed that PTBP1 KD-mediated apoptosis inhibition confers robust neuroprotection against SPS-induced hippocampal neuronal loss. In addition, PTBP1 not only regulates the expression of Bcl-2 protein family members, but also participates in the regulation of the expression of apoptosis or necrosis regulators such as RIPK1 and Fas, which often plays a promoting role in the process of neuronal loss^45,46^. In conclusion, inhibition of apoptosis by downregulation of PTBP1 is an effective approach to rescue hippocampal neuron loss in PTSD mice.

During the process of neuronal differentiation leading to maturity, it can be broadly divided into three stages: neurite outgrowth, dendrite elaboration and axon elongation, as well as synaptogenesis. This progression of changes highly coincides with the alterations in the expression of PTBP1 and BTBP2^47^. Notably, synaptic plasticity enhancement emerged as a therapeutic avenue for alleviating PTSD-associated behavioral deficits^48,49^. PSD95 is essential for synaptic development and neuronal differentiation, with its exon 18 undergoing joint suppression by PTBP1 and PTBP2. The activation of exon 18 splicing occurs specifically during later stages of neuronal development, following the downregulation of PTBP2 expression^24^. In this study, the impact of hippocampal PTBP1 KD on synaptic plasticity and axonal regeneration was examined in PTSD mice. Our findings reveal that PTBP1 KD in the hippocampus of PTSD mice elevated PSD95 and SYN1 expression levels, accompanied by a pronounced increase in dendritic spine density. These outcomes suggest that PTBP1 downregulation enhances synaptic plasticity in the PTSD mouse hippocampus. Notably, PTBP1 KD suppressed alternative splice site selection within SynGAP1 intron 10 while enhancing SynGAP1 transcription, indicating that this regulatory mechanism may further support synaptic plasticity enhancement^10^. However, our research suggests that PTBP1 KD does not entirely promote synaptic plasticity. In fact, synaptic plasticity is dependent on dendrites and dendritic spines. Dendrites indirectly participate in the formation and regulation of dendritic spines, thereby influencing synaptic plasticity. In our study, while PTBP1 promoted the growth of dendritic spines, it did not exhibit significant positive effects on dendritic morphology or such beneficial effects may have been masked by concurrent negative outcomes. Moreover, in normal mice, KD of PTBP1 alone was sufficient to suppress dendritic growth and arborization. Our research supports the notion that downregulation of PTBP1 activates the Nogo-A/NgR/RhoA/ROCK2 axis, where RhoA is generally recognized as a negative regulator of dendrites. The activation of RhoA/ROCK2 is detrimental to dendrite growth and remodeling^50,51^. This may be the reason why downregulation of PTBP1 inhibits the state of dendrites.

In this study, PTBP1 KD upregulated NF200 expression but failed to elevate GAP-43 levels, suggesting that PTBP1 KD exerts both promotive and inhibitory effects on neuronal axon regeneration capacity. Currently, there is limited research elucidating whether downregulation of PTBP1 in mature neurons inhibits axon regeneration. However, a recent study found that PTBP1 is expressed in mature peripheral nerve axons and is significantly upregulated after injury, participating in the regulation of injury response and the expression of factors related to axon regeneration, while inhibiting the translation of RhoA^52^. This indicates that PTBP1 has specific functions in mature peripheral neurons or neuronal subtypes, particularly in promoting axon growth. However, this study did not clarify whether PTBP1 exerts similar effects in the mature CNS^52^. Our results show convergence with the findings of the study of Alber and colleagues^52^. While their study established PTBP1’s role in regulating the axonal local translation of mRNAs, including that of RhoA (a process crucial for neuronal maintenance and regeneration), our work provides direct in vivo evidence within the CNS. Specifically, we demonstrate that PTBP1 KD alone activates the Nogo-A/NgR/RhoA/ROCK2 inhibitory pathway, leading to impaired axonal regeneration and dendritic abnormalities in both normal mice and PTSD models. Moreover, we present the evidence in normal animals that such molecular impairments translate directly into behavioral deficits, notably manifesting as anxiety-like behavior.

The results of this study support that PTBP1 KD upregulates Nogo-A and activates the NgR/RhoA/ROCK2 axis, consistent with previous findings^33,34^. This was shown to cause neuronal axonal aplasia, indicating that KD of PTBP1 negatively affects neuronal axonal regeneration^53^. However, there remains a need for more rigorous evidence to substantiate the presence of PTBP1 in mature neurons of CNS, as well as to demonstrate that the aforementioned effects are specifically achieved through knocking down PTBP1 in CNS neurons, rather than in other cells. We propose that this phenomenon can be explained by a mechanism resembling the physiological downregulation of PTBP1. PTBP1 expression is sharply downregulated and PTBP2 expression is transiently increased before and after neural stem cells exit mitosis, and the changes in their expression promote neuronal differentiation and maturation^13,14,24,34,47,54,55^. PTBP1 KD turns on neuronal gene expression changes, such as splicing regulation of PTBP2, Pbx1, Dpf2 and DNMT3B^8,9,20^. PTBP2 alternative splicing regulates the expression of Shtn1. Transient upregulation of PTBP2 promotes the expression of Shtn1s isoform and promotes neurite formation, while re-downregulation of PTBP2 increases the expression of Shtn1l isoform and promotes axon elongation and synapse formation^32^. Notably, our results demonstrated marked elevation of RhoA levels following SPS exposure or PTBP1 KD, with particularly pronounced induction observed after PTBP1 KD. Acting as a key effector of NgR signaling, RhoA’s established role in mediating Nogo-induced suppression of axonal regeneration has been extensively characterized. Paradoxically, recent studies demonstrated divergent functional outcomes of RhoA activity across neuronal and astrocytic populations^53^. RhoA inhibits astrocyte reactivity by activating type II myosin-mediated actin to promote neuronal axonal regeneration. Loss of astrocyte RhoA after spinal cord injury enhances astrocyte reactivity and inhibits neuronal axonal regeneration by activating Yes-associated protein (YAP), a key factor in actin cytoskeleton regulation^53^. This implies that both promoting and inhibiting effects on axon regeneration after PTBP1 KD can be attributed to upregulation of RhoA. In summary, the outcome of our observed effects of PTBP1 KD on axon regeneration is a superimposition of promoting and inhibitory influences, which contribute to the structural stabilization of axons while disadvantaging their elongation.

Based on the aforementioned research, we further explored approaches to counteract the inhibitory effects of PTBP1 KD on synaptic plasticity and axon regeneration. Nogo-A, the most potent inhibitor of axon regeneration within the Nogo family, is upregulated following PTBP1 KD, further mediating the activation of the NgR/RhoA/ROCK2 pathway to suppress synaptic plasticity and axon regeneration. The 66-residue Nogo-66 domain within Nogo-A is expressed in oligodendrocytes and binds directly to the NgR receptor. Through interaction with the coreceptor p75NTR, this molecular complex activates RhoA signaling, ultimately inducing growth cone collapse and suppressing neurite extension^32,56,57^. In previous studies, various inhibitors or competitive antagonists were used to block the activation of this pathway and promote axonal growth at different points along it^58,59^. Nevertheless, p75NTR has multiple functions and a wide influence range, while RhoA expression is extensively distributed and lacks specificity. Hence, it is not appropriate as an intervention target. NgR is a potential intervention target due to its potent effect and high neural specificity^57,60^. Based on this, in the current research, we chose to KD NgR to inhibit the activation of the RhoA-ROCK2 pathway, which effectively enhanced dendrite growth and axonal regeneration capabilities in PTSD mice. When PTBP1 and NgR were knocked down simultaneously, it effectively reversed the inhibition of dendrite growth and axonal regeneration caused by PTBP1 KD, thereby facilitating axonal regeneration and synaptic plasticity more effectively. Similarly, in normal mice, the combined downregulation of PTBP1 and NgR rescued the axonal regeneration deficits and anxiety-like behavior induced by PTBP1 KD alone. Notably, whether downregulation of PTBP1 can rescue neuronal loss through other pathways—such as promoting stem cell differentiation into neurons or transdifferentiation of glial or other cells into neurons—and whether it may cause additional harm to neurons remains to be investigated.

This study supports the therapeutic potential of PTBP1 as a PTSD treatment target, with accumulating evidence from multiple experimental approaches. Our findings establish that multigene regulatory approaches represent a critical component of effective therapeutic design. Rigorous functional genomic investigations must prioritize comprehensive risk–benefit analysis, particularly when examining genes such as PTBP1 that exert pleiotropic regulatory control over neural systems. Failure to adopt this balanced approach risks undermining the translational validity of gene-based interventions.

In conclusion, PTBP1 KD reduced hippocampal neuronal loss through apoptosis inhibition, but it simultaneously caused adverse effects on synaptic plasticity and axon regeneration in hippocampal neurons. This negative effect appears to be a general characteristic, independent of specific pathological contexts. The combined KD of PTBP1 and NgR demonstrated greater therapeutic potential in alleviating PTSD-like behavioral deficits. Its primary advantages may lie in mitigating the adverse effects associated with PTBP1 KD and inducing more comprehensive improvements in molecular mechanisms related to axonal regeneration and synaptic plasticity.

## Data Availability

Data will be made available on reasonable request.
